# Dry Olive Leaf Extract Counteracts L-Thyroxine-Induced Genotoxicity in Human Peripheral Blood Leukocytes *In Vitro*

**DOI:** 10.1155/2015/762192

**Published:** 2015-02-19

**Authors:** Dijana Žukovec Topalović, Lada Živković, Andrea Čabarkapa, Ninoslav Djelić, Vladan Bajić, Dragana Dekanski, Biljana Spremo-Potparević

**Affiliations:** ^1^Department of Biology and Human Genetics, Institute of Physiology, Faculty of Pharmacy, University of Belgrade, Vojvode Stepe 450, 11000 Belgrade, Serbia; ^2^Department of Biology, Faculty of Veterinary Medicine, University of Belgrade, Bulevar Oslobođenja 18, 11000 Belgrade, Serbia; ^3^The Laboratory for Radiobiology and Molecular Genetics, Institute for Nuclear Research “Vinča”, University of Belgrade, Mike Petrovića Alasa 12-14, 11000 Belgrade, Serbia; ^4^Biomedical Research, R&D Institute, Galenika a.d., Pasterova 2, 11000 Belgrade, Serbia

## Abstract

The thyroid hormones change the rate of basal metabolism, modulating the consumption of oxygen and causing production of reactive oxygen species, which leads to the development of oxidative stress and DNA strand breaks. Olive (*Olea europaea* L.) leaf contains many potentially bioactive compounds, making it one of the most potent natural antioxidants. The objective of this study was to evaluate the genotoxicity of L-thyroxine and to investigate antioxidative and antigenotoxic potential of the standardized oleuropein-rich dry olive leaf extract (DOLE) against hydrogen peroxide and L-thyroxine-induced DNA damage in human peripheral blood leukocytes by using the comet assay. Various concentrations of the extract were tested with both DNA damage inducers, under two different experimental conditions, pretreatment and posttreatment. Results indicate that L-thyroxine exhibited genotoxic effect and that DOLE displayed protective effect against thyroxine-induced genotoxicity. The number of cells with DNA damage, was significantly reduced, in both pretreated and posttreated samples (*P* < 0.05). Comparing the beneficial effect of all tested concentrations of DOLE, in both experimental protocols, it appears that extract was more effective in reducing DNA damage in the pretreatment, exhibiting protective role against L-thyroxine effect. This feature of DOLE can be explained by its capacity to act as potent free radical scavenger.

## 1. Introduction

Triiodothyronine (T_3_) and thyroxine (tetraiodothyronine, T_4_), produced by the thyroid gland, play a role in the regulation of growth, development, and differentiation [1]. It is well known that the thyroid hormones (THs) stimulate the metabolism of cells and tissues, regulating the consumption of oxygen. However, it is also known that by increasing aerobic metabolism in mitochondria, they can cause intense production of reactive oxygen species (ROS) and reactive nitrogen species (RNS), leading to the condition of oxidative stress which is involved in the pathogenesis of many human diseases, including age-related chronic diseases, cancer, muscle degeneration, and coronary heart disease [2–5]. On the other hand, THs also affect the cell antioxidant mechanisms [6]. The evidence available shows a complex relationship between levels of thyroid hormones and oxidative stress, but the general principle is that elevated TH levels (hyperthyroidism) induce oxidative stress, whereas reduced THs levels (hypothyroidism) result in nondetectable to mild oxidative stress [6, 7]. In addition to lipid peroxidation, elevated level of thyroid hormones promotes protein oxidation in rat liver [8] as well as in human leukocytes [9]. There are some indications that phenolic groups of nonsteroid hormones, such as adrenaline and thyroid hormones, and of neurotransmitters dopamine and noradrenaline increase the endogenous formation of reactive oxygen species, leading to oxidative stress and DNA strand breaks [10–13]. Furthermore, some experimental findings confirm that THs induced DNA damage in sperm cells [12] and human lymphocytes [11].

Many single components of traditional Mediterranean diet are known to have positive effects on health, reducing oxidative stress, inflammation, and other important risk factors of age-related diseases. The research of pharmacological properties of the bioactive components of this dietary pattern is very active and could lead to the formulation of functional foods and nutraceuticals [14]. A key role is played by polyphenols presented in high amount, in particular in extra virgin olive oil [15, 16]. In humans, recent clinical trial has reported that the daily consumption (50 mL) of oleuropein-rich extra virgin olive oil increased the total antioxidant capacity in plasma of healthy elderly people [17]. The results of the same study also show a significant increase of catalase in erythrocytes and a decrease in superoxide dismutase and glutathione peroxidase activity [17]. Due to biological effects of its phenolic compounds, olive mill wastewater extract is already suggested for inclusion into food and beverages [18]. Olive (*Olea europaea* L.) leaf contains large amounts of potentially useful phytochemicals, many of the same phenolics as the olive oil but in much higher concentration [19, 20]. The main constituent of olive leaf extract is oleuropein, one of iridoid monoterpenes. Olive leaf also contains triterpenes (oleanolic, ursolic, and maslinic acid), flavonoids (luteolin, apigenin, and quercetin), caffeic acid, and tannins [21, 22]. Its chemical content makes olive leaf one of the most potent natural antioxidants [23]. The beneficial properties of olive leaf are further enhanced by the good absorption of its active constituents and their bioavailability, which is a necessary precondition for its activity against oxidative stress-related processes* in vivo* [24]. A number of studies have reported the beneficial physiological and pharmacological properties of oleuropein [25]. One of its most prominent properties is its strong antioxidant activity, mainly due to the presence of hydroxyl groups in its chemical structure which could donate hydrogen to prevent oxidation. In our previous study, high antioxidative potential of the oleuropein-rich, standardized dry olive leaf extract (DOLE) was confirmed* in vitro* using the 2,2-diphenyl-1-picrylhydrazyl (DPPH^•^) [26]. Our additional findings have suggested its strong antioxidative potential* in vivo*, in different experimental models: in ethanol- and cold restraint stress-induced gastric ulcers [27, 28], in global cerebral ischemia and reperfusion [26], and in spontaneously hypertensive rats [29]. In all these experimental studies, DOLE strongly influenced lipid peroxidation and antioxidative enzyme activity in different tissues. Furthermore, the results of recent study show that treatment with olive leaf extract caused decrease in tissue malondialdehyde, diene conjugate and protein carbonyl levels, and increased hepatic glutathione levels in aged rats [30].

Although different* in vitro* systems have shown that olive phenolics possess a potent antioxidative activity and prevent the ROS-mediated cell injury, there is limited evidence for such protective role on DNA damage. Fabiani et al. [31] have demonstrated that olive phenolics, when used both as purified compounds and in complex crude extracts and regardless of the source (olive oil or olive mill wastewater), may prevent the H_2_O_2_-induced and phorbol myristate acetate-induced DNA damage in a very low concentration range. In another study, the Somatic Mutation and Recombination Test in wing imaginal discs of* Drosophila melanogaster* has been performed to test the possible genotoxicity of total olive leaf extract and the individual components oleuropein and luteolin. None of the extracts or phenols tested showed significant mutagenic activity. This fact, together with the antigenotoxic activity against H_2_O_2_, detected for total extract or its constituents, confirmed the safety of olive leaf, oleuropein, and luteolin in terms of DNA protection [32]. The protective effects of olive leaf extract on genotoxicity and oxidative damage in cultured human blood cells treated with permethrin (a highly toxic synthetic pyrethroid pesticide) were found recently [33]. Nevertheless, olive leaf extract is much less studied for its ability to reduce oxidative DNA damage induced by hormones. We have recently demonstrated the beneficial effects of DOLE in adrenaline-induced genotoxicity [34].

The objective of this study was to evaluate the genotoxicity of L-thyroxine and to investigate antioxidative and antigenotoxic potential of the standardized dry olive leaf extract against L-thyroxine-induced DNA damage in human peripheral blood leukocytes by using the comet assay: “single cell gel electrophoresis.” The comet assay has been shown to be an effective, sensitive, and rapid* in vitro* method for examining DNA damage and issues related to oxidative stress in human peripheral blood cells [35].

## 2. Material and Methods

### 2.1. Subjects

Peripheral blood samples from six healthy volunteers (6 female subjects) aged between 20 and 39 years were collected in heparinized containers. Volunteers were nonsmokers and did not consume alcohol, receive any therapy and medications, or take dietary supplements.

### 2.2. Olive Leaf Extract

Olive leaf extract EFLA 943, standardized to 18–26% of oleuropein, was purchased from Frutarom Switzerland Ltd. (Wadenswil, Switzerland). The extract was manufactured from the dried leaves of* Olea europaea* L., applying an ethanol extraction procedure. Stability and microbiological purity were confirmed by the manufacturer. The comprehensive phytochemical analysis was performed previously, using high performance liquid chromatography [21]. In this study, the same batch of EFLA 943 was used. It was kept in sealed microtubes, stored at room temperature, and protected from light until use.

### 2.3. Study Design

For this study, L-thyroxine sodium salt pentahydrate (T_4_) (CAS no. 6106-07-6, Galenika, Belgrade, Serbia) was used. According to literature data, the concentration of L-thyroxine of 50 *μ*M caused a significant decrease in mitotic index [36]. Also, thyroid hormones in a range 10–100 *μ*M caused DNA damage in the comet assay [11, 12]. The hormone was preexamined at several concentrations (data not shown in the results) and 50 *μ*M thyroxine was chosen for further testing in our study, since this was the concentration which produced significant level of DNA damage in treated cells, but also retained good cell viability. For our study design, DOLE powder was diluted in phosphate buffered saline (PBS, Torlak Institute of Immunology and Virology, Belgrade, Serbia) to three final concentrations: 0.125 mg/mL, 0.5 mg/mL, and 1 mg/mL. Final concentrations of the extract were determined based on the range of concentrations documented to be safe and effective in* in vitro* experiments [33, 34, 37, 38]. In order to evaluate ability of commercial DOLE to prevent nuclear DNA in human peripheral blood leukocytes from the damage induced by L-thyroxine, we performed two types of interactions of DOLE extract with the oxidant: pretreatment and posttreatment. In the first series (pretreatment protocol), three different concentrations of DOLE (0.125, 0.5, and 1 mg/mL) were administered and incubated with peripheral blood leukocytes preparations at 37°C for 30 min and rinsed with PBS before adding T_4_ for 30 min on 37°C. In the second series (posttreatment protocol), cell preparations from same individuals were firstly exposed to oxidant, then washed with PBS, and posttreated with same concentrations of DOLE as in pretreatment. Since hydrogen peroxide (H_2_O_2_) is one of the most studied chemicals known to produce ROS and induce oxidative stress, H_2_O_2_ was used as the inducer of the DNA damage in the second experiment. H_2_O_2_ was added for 15 min on ice to induce DNA damage with other conditions, the same as described above. This was performed in order to assess effect of DOLE on different inducers of DNA damage. 25 *μ*M hydrogen peroxide (CAS no. 7722-84-1, ZORKA Pharma, Sabac, Serbia) was used for testing. Negative controls were treated only with PBS. We performed experiments for all six samples, each in duplicate.

### 2.4. The Single Cell Gel Electrophoresis Assay

**Figure 1:**
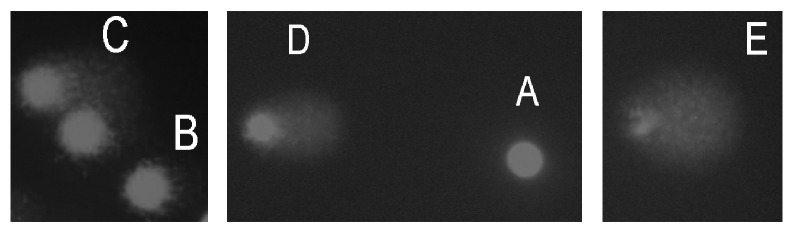
Five categories of comets: (A) no damage, <5%; (B) low level damage, 5–20%; (C) medium level damage, 20–40%; (D) high level damage, 40–95%; (E) total damage, >95%.

Before both treatments, cell viability for all samples was determined by using the trypan blue exclusion method [39] and it was found to be above 90% for all samples. The comet assay was performed as described by Singh et al. [40]. 6 *μ*L of whole blood samples was first suspended in 0.67% low-melting-point (LMP) agarose (Sigma-Aldrich, St. Louis, MO) and pipetted onto superfrosted glass microscope slides. Slides were previously coated with a layer of 1% of normal-melting-point agarose (Sigma-Aldrich, St. Louis, MO) and maintained to solidify for 5 min on 4°C. After gently removing the cover slips placed over the layer of agarose, the cell suspensions on slides were treated with DOLE and oxidants as described, under two protocols, pretreatment and posttreatment. Following the treatments, all slides were covered with the third layer of 0.5% LMP agarose and kept once again on ice for 5 min to solidify. After removing the cover slips, slides were submerged in previously prepared and cooled lysing solution (2.5 M NaCl, 100 mM EDTA, 10 mM Tris, 1% Triton X100, and 10% dimethylsulfoxide, pH 10 adjusted with NaOH) and left to stay at 4°C overnight. The next day, slides were subjected to electrophoresis. After the electrophoresis, the slides were rinsed with neutralization buffer and distilled water. This was done three times with neutralization buffer and afterwards one time with distilled water and each time the slides were allowed to stand for 5 minutes. Slides were than stained with ethidium bromide (20 *μ*g/L). 15 min after staining, the comets were analyzed at magnification of 100x on Olympus BX 50 microscope (Olympus Optical Co., GmbH, Hamburg, Germany), equipped with a mercury lamp HBO (50 W, 516–560 nm, Zeiss). DNA damage was evaluated according to Anderson et al. [39]. DNA damage in the cells was assessed by quantification of the amount of DNA released from the core of the nucleus and comets were visually scored and classified into five categories corresponding to the extent of DNA migration: (A) no damage, <5%; (B) low level damage, 5–20%; (C) medium level damage, 20–40%; (D) high level damage, 40–95%; (E) total damage, >95% (Figure 1). Since treatments for each of 6 subjects were done in duplicate, analysis was performed on 50 randomly selected cells on each of 2 slides per subject (100 cells in total per subject) and was always carried out by the same experienced person. DNA damage was characterized as DNA migration over 5% (B + C + D + E comet classes), and the mean and standard error (SEM) was calculated for all six subjects. Leukocytes undergoing apoptosis or necrosis were excluded from the analysis and separated from normal cells following the instructions given by Singh [41].

### 2.5. Statistical Analysis

The results were obtained as the mean and standard error (SEM), for *n* = 6. In all experiments, the data were analyzed for statistical significance using analysis of variance (one-way ANOVA) with Tukey's post hoc test. All data were analyzed with the GraphPad Prism 5.0 software. A difference at *P* < 0.05 was considered statistically significant.

## 3. Results

**Figure 2:**
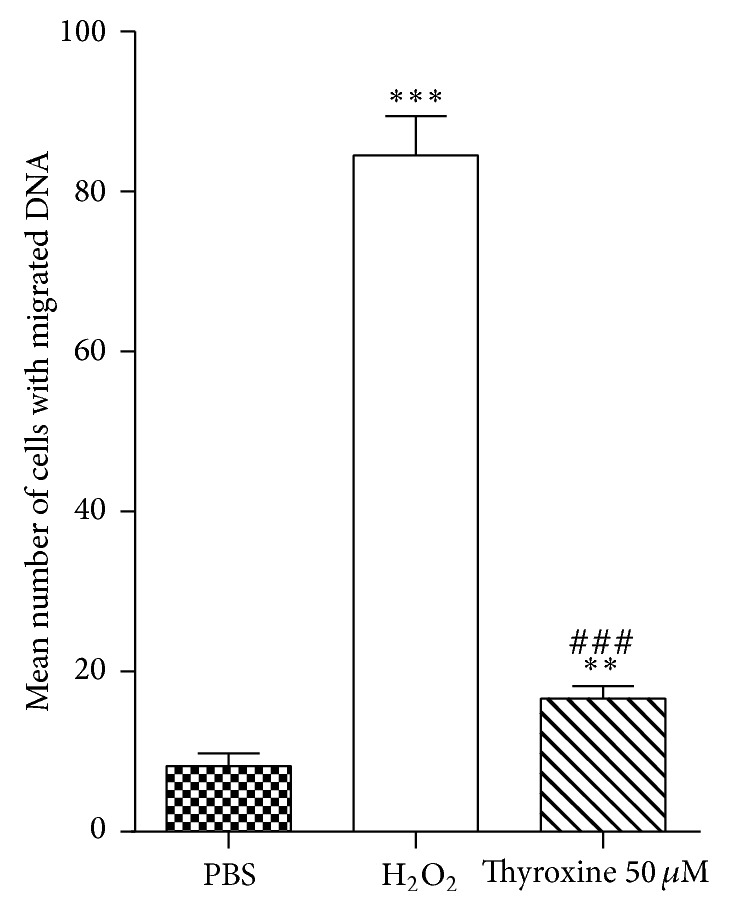
Degree of DNA damage in human leukocytes exposed to thyroxine and H_2_O_2 _treated positive control separately, compared to negative control treated with PBS. ^**^ *P* < 0.01, ^***^ *P* < 0.001 thyroxine and H_2_O_2_ treatment versus PBS; ^###^ *P* < 0.001 thyroxine versus H_2_O_2_ by Mann Whitney test. Data represent mean ± SEM from 6 subjects.

The genotoxic effect of 50 *μ*M thyroxine is shown in Figure 2, represented as the mean number of cells with damaged DNA (±standard error) compared to the effect of 25 *μ*M H_2_O_2_ as positive control and PBS treated cells as negative control (*P* < 0.01). Increase of DNA damage was detected in leukocytes exposed to thyroxine and H_2_O_2_, compared to negative control. Thyroxine was able to cause DNA strand breaks in more than 15% of the treated cells, compared to over 80% of DNA damaged cells in positive control and 8% for negative control.

Protective ability of DOLE was tested using pretreatment and posttreatment protocols and the mean number of DNA damaged cells was compared to the mean number obtained for the DNA damage induced by H_2_O_2_ or by L-thyroxine. The mean number of cells for the effect of pretreatment with DOLE on the level of DNA damage is shown in Table 1. Values represent mean numbers of DNA damaged cells preincubated with DOLE and afterwards exposed to oxidant. It is evident that the extract exhibited protective effect at all concentrations. Also, as shown in Table 1, for the effect of pretreatment with DOLE, there is no concentration dependence between the level of DNA damage induced by thyroxine and the concentration of DOLE. In this case, dry olive leaf extract was most effective in attenuating the number of cells with DNA damage at the highest concentration, 1 mg/mL (*P* < 0.03), while two smaller concentrations of the extract (0.5 and 0.125 mg/mL) showed approximate results. An inverse response can be seen between the level of DNA damage and the concentration of DOLE in cells exposed to hydrogen peroxide. Here, the levels of DNA damage in analyzed cells decline with the decrease of DOLE concentration, meaning that DOLE was most effective at 0.125 mg/mL.

The results for cells subjected to posttreatment protocol are shown in Table 2. These cells were first exposed to thyroxine and afterwards posttreated with antioxidant, DOLE, under the same conditions as described above. It is evident that the extract showed the strongest effect at 0.5 mg/mL and that there was no concentration dependence. Interestingly, a U shape or biphasic dose- (concentration-) response relationship between the concentration of DOLE and the level of nuclear DNA damage induced by L-thyroxine and H_2_O_2_ is also evident. The results in Tables 1 and 2 show that the number of cells with DNA damage, from the samples treated with different concentrations of DOLE were significantly reduced, both in pretreated and posttreated samples (*P* < 0.05). All concentrations of DOLE that were used (0.125, 0.5, and 1 mg/mL) strongly reduced the number of cells with damaged DNA under both experimental protocols with both DNA damage inducers. Comparing the antigenotoxic effect of all concentrations of dry olive leaf extract, in both pretreatment and posttreatment protocols, it appears that extract was more effective in reducing DNA damage in pretreatment than in posttreatment, exhibiting protective ability against L-thyroxine effect.

## 4. Discussion

Some pathological conditions can alter the level of thyroid hormones, changing the rate of basal metabolism and leading to the development of oxidative stress [4, 6, 7]. It is generally accepted that oxidative stress affects the organism when the generation of ROS exceeds the capacity of the cells to reduce them. Increased oxidative stress induces damage on proteins, membrane lipids, and DNA. A wide range of studies focus especially on substances causing oxidative DNA damage. The fact that the THs affect various aspects of the oxidative stress could explain the inconsistencies in the literature on the effects of thyroid hormones [6] and some discordant results regarding the genotoxic activity of thyroid hormones. While protein damage was already shown after short-term exposure to thyroxine, oxidative DNA damage in mouse liver and heart was not found even after longer-term treatment [7]. Some explanations can be provided as the lack of increase of one of the major products of DNA oxidation, 8-oxo-dG, after short-term treatment. Firstly, it is probable that most of the H_2_O_2_ generated by various cellular sources is removed by antioxidants in the cytosol before it reaches the nucleus. Secondly, nuclear DNA is extensively covered by histones, which make it less susceptible to ROS. Finally, 8-oxo-dG is rapidly repaired by specific enzymes and increase of oxidative stress enhances the repair of nuclear DNA oxidative damage during thyroid hormone-induced oxidative stress [7]. Indeed, evaluation of the possible clastogenic effects of L-thyroxine on the cultured whole blood lymphocytes showed no statistically significant structural chromosome aberrations and, only at the highest experimental concentration, was able to significantly decrease mitotic index [36, 42]. On the other hand, it has been documented that excessive amounts of the free thyroid hormones triggered hypermetabolic state* in vivo*, with subjects exhibiting low antioxidant capacity and high susceptibility to oxidative challenge with excess generation of free radicals [43]. Also, alteration in the thyroid function by T_4_ influences the antioxidant defense system, indicating that excess thyroxine has stress generating effects on liver and other organs [5]. Although studies on the evaluation of the oxidative stress induced by thyroxine* in vitro* using the comet assay are sparse, there are some results showing that thyroid hormones induced an increasing DNA damage in human sperm cells [12] and in human leukocytes* in vitro* [11, 36]. The aforementioned results provide strong evidence that THs induce oxidative stress in target cells. Results presented in this study confirm that L-thyroxine is able to cause DNA strand breaks in human peripheral blood leukocytes using the comet assay methodology, in more than 15% of the treated cells. Although we used exposure time period of only 30 minutes, the hormone exhibited genotoxic effect.

It is confirmed that some antioxidants, such as vitamin E and curcumin, are efficient in protecting sperm cells from oxidative stress generated by T_4_, mainly by restoring antioxidant enzymes to the level of euthyroid animals [44]. Dry olive leaf extract is one of the antioxidants able to decrease the number of cells with nuclear DNA damage induced by oxidizing agents [34]. DOLE has the total content of phenols of 197.8 *μ*g GAE/g, total content of flavonoids 0.29%, and tannins 0.52%. High performance liquid chromatography (HPLC) analysis showed a complex mixture of phenolic and flavonoid compounds: oleuropein (19.8%), luteolin-7-O-glucoside (0.04%), apigenin-7-O-glucoside (0.07%), quercetin (0.04%), and caffeic acid (0.02%) [21]. To the best of our knowledge, this is the first study to examine phenomenon of thyroid hormone induced DNA damage being inhibited by the extract of olive leaf.

Our study confirms that* Olea europaea* leaf extract has the potential to reduce oxidative DNA damage in human leukocytes induced by thyroxine under two different experimental conditions, pretreatment and posttreatment. Results of the pretreated samples showed that the highest concentration of the extract (1 mg/mL) exhibited the highest decrease in the number of cells with DNA damage in the samples treated with L-thyroxine. Efficiency of pretreatment with the extract against thyroxine-induced DNA damage can be explained by the mechanisms reported by some authors that DOLE increased the cells' antioxidant capacity by stimulating the synthesis of antioxidant enzymes and helped maintain their activity during oxidative stress [45]. On the contrary, in the pretreatment with DOLE against H_2_O_2_, the smallest concentration of extract used (0.125 mg/mL) actually corresponded to the smallest number of cells with DNA damage. The effect of concentrations of DOLE, 1 mg/mL for thyroxine and 0.125 mg/mL for H_2_O_2_, can be ascribed to the different type of DOLEs mechanisms of action with L-thyroxine and H_2_O_2_.

The positive effects of posttreatment with DOLE were different from the ones in pretreatment. The highest concentration of DOLE (1 mg/mL) displayed the lowest protective effect on leukocytes observed in posttreatment, compared to two smaller concentrations of the extract, with both thyroxine and H_2_O_2_. Another phenomenon, known as hormesis, can be observed from the results of posttreatment protocol, since the extract was most effective at 0.5 mg/mL. Hormesis is a biphasic dose response to an environmental agent characterized by low dose stimulation or beneficial effect and a high dose inhibitory or toxic effect. It is defined as an adaptive response of cells and organisms to a moderate stress [46]. These quantitatively similar U-shaped dose responses have long been recognized by researchers as being involved with factors affecting the oxidative stress mediated degenerative responses and that dietary polyphenols also act hermetically, displaying cytoprotective effects at low doses [47].

The protective effect of DOLE is probably a result of synergistic activation of several mechanisms such as ROS scavenging and stimulation of DNA repair. The applications of different protocols with the extract made it possible to assess the mechanisms involved in DOLE antigenotoxic effects. It should also be mentioned that genotoxic properties of the extract were evaluated in an independent experiment in our previous research and that DOLE did not cause DNA damage at any of the concentrations used in this study [34]. As already stated, main components of DOLE are phenolics and flavonoids. Both the individual and combined phenolics from extract exhibited good free radical scavenging abilities. The effect of the combined phenolics was significantly higher than that of the individual constituents [23, 48]. Other findings have confirmed that flavonoids, kaempferol, and quercetin, other important constituents of DOLE, produced inhibitory responses to the induced DNA damage [12]. Moreover, it was shown that olive leaf extract had a higher antioxidant activity than vitamin C and vitamin E, due to the synergy between the flavonoids, oleuropeosides, and substituted phenols [23]. Consequently, since different phenolic components show synergistic behavior for maximal radical scavenging capacity, DOLEs positive effect can also be explained by its capacity to act as potent free radical scavenger. Further cytochemical detection of stimulated superoxide generation sites could provide the useful additional information, and our future investigation will be focused on the antioxidant properties of DOLE during the experimental conditions we used in this study.

Based on our results, it can be said that DOLE was efficient under both experimental conditions, pretreatment and posttreatment, but more effective in reducing oxidative DNA damage in the pretreatment. Positive results in both protocols indicate that olive leaf extract has genoprotective feature in the thyroxine-induced genotoxicity and can be considered a potential candidate to protect all cells against the deleterious effect of oxidative DNA damages in hyperthyroid state. Furthermore, this* in vitro* evaluation has also provided interesting results that might be beneficial for the use of DOLE in* in vivo* experimental studies as well as in future clinical trials.

## 5. Conclusions

L-Thyroxine was able to cause DNA damage in human peripheral blood leukocytes and exhibit genotoxic effect. Dry olive leaf extract was efficient in reducing DNA damage induced by thyroxine at all tested concentrations used in our study and under both experimental protocols, pretreatment and posttreatment. Therefore, based on our findings using the comet assay methodology, we can conclude that dry olive leaf extract has DNA protective ability.

## Figures and Tables

**Table 1:** Pretreatment protocol: number of cells with damaged DNA from six different subjects, pretreated with different concentrations of dry olive leaf extract (DOLE) and subsequently exposed to oxidants (thyroxine and H_2_O_2_).

Oxidants		DOLE concentrations		
		1 mg/mL	0.5 mg/mL	0.125 mg/mL
Thyroxine (50 *µ*M)	16.67 ± 1.54	3.58 ± 1.03^*^	4.42 ± 1.16^*^	4.25 ± 1.09^*^
H_2_O_2 _(25 *µ*M)	84.5 ± 4.93	20 ± 7.69^*^	9.83 ± 1.77^*^	8.5 ± 1.94^*^

Values are expressed as mean ± SEM for comet scores in 100 cells from 6 subjects.

^*^ *P* < 0.05 DOLE treatment versus oxidant, analyzed by one-way ANOVA test of variance.

**Table 2:** Posttreatment protocol: number of cells with damaged DNA from six different subjects, first treated with thyroxine and H_2_O_2_ and subsequently incubated with different concentrations of dry olive leaf extract (DOLE).

Oxidants		DOLE concentrations		
		1 mg/mL	0.5 mg/mL	0.125 mg/mL
Thyroxine (50 *µ*M)	16.67 ± 1.54	6.92 ± 1.24^*^	3.58 ± 0.68^*^	5.25 ± 0.72^*^
H_2_O_2 _(25 *µ*M)	84.5 ± 4.93	16.83 ± 3.94^*^	6.83 ± 2.70^*^	14.5 ± 4.31^*^

Values are expressed as mean ± SEM for comet scores in 100 cells from 6 subjects.

^*^ *P* < 0.05 DOLE treatment versus oxidant, analyzed by one-way ANOVA test of variance.
